# The two coiled-coil domains of the fission yeast kinesin-6 Klp9 are required for motor tetramerization and spindle elongation

**DOI:** 10.17912/micropub.biology.001829

**Published:** 2025-09-22

**Authors:** Mason T Nguyen, Liang Ji, Phong T Tran

**Affiliations:** 1 Corona del Mar High School, Newport Beach, CA 92660, USA; 2 Institut Curie, PSL Université, Sorbonne Université, CNRS UMR 144, Paris 75005, France; 3 University of Pennsylvania, Department of Cell & Developmental Biology, Philadelphia, PA 19104, USA

## Abstract

The fission yeast kinesin-6 Klp9 localizes to the spindle midzone at anaphase to produce sliding forces to elongate the bipolar spindle. In the absence of Klp9 , anaphase spindle elongation is attenuated by half its normal rate. Klp9 functions as a microtubule plus end-directed tetrameric motor. Tetramerization is the key to its microtubule sliding function, as tetramerization allows Klp9 to bind antiparallel microtubules at the midzone. The amino acid sequence of Klp9 indicates two alpha-helical coiled-coils domains CC1 and CC2, important for protein-protein interactions. We seeked the potential oligomerization states of Klp9 via its coiled-coils using AlphaFold3. AlphaFold predicted that CC1 can form dimers and together with CC2 can form tetramers. The different oligomeric states enabled precise experimental verifications. We measured Klp9 motor GFP intensity and anaphase spindle elongation rate for the full-length Klp9 , Klp9-deletion (Klp9Δ), and truncated Klp9 containing no coiled-coils, or only CC1, or both CC1 and CC2. The results indicate that: 1) GFP intensity increases with increasing oligomeric state, and 2) attenuated anaphase spindle velocity is restored only in the Klp9 truncation containing both CC1 and CC2. The experimental data are consistent with prediction, indicating that CC1 contributes to Klp9 dimerization, and that CC1 and CC2 together contribute to Klp9 tetramerization.

**Figure 1. Coiled-coils of kinesin-6 Klp9 are required for oligomerization and function:**
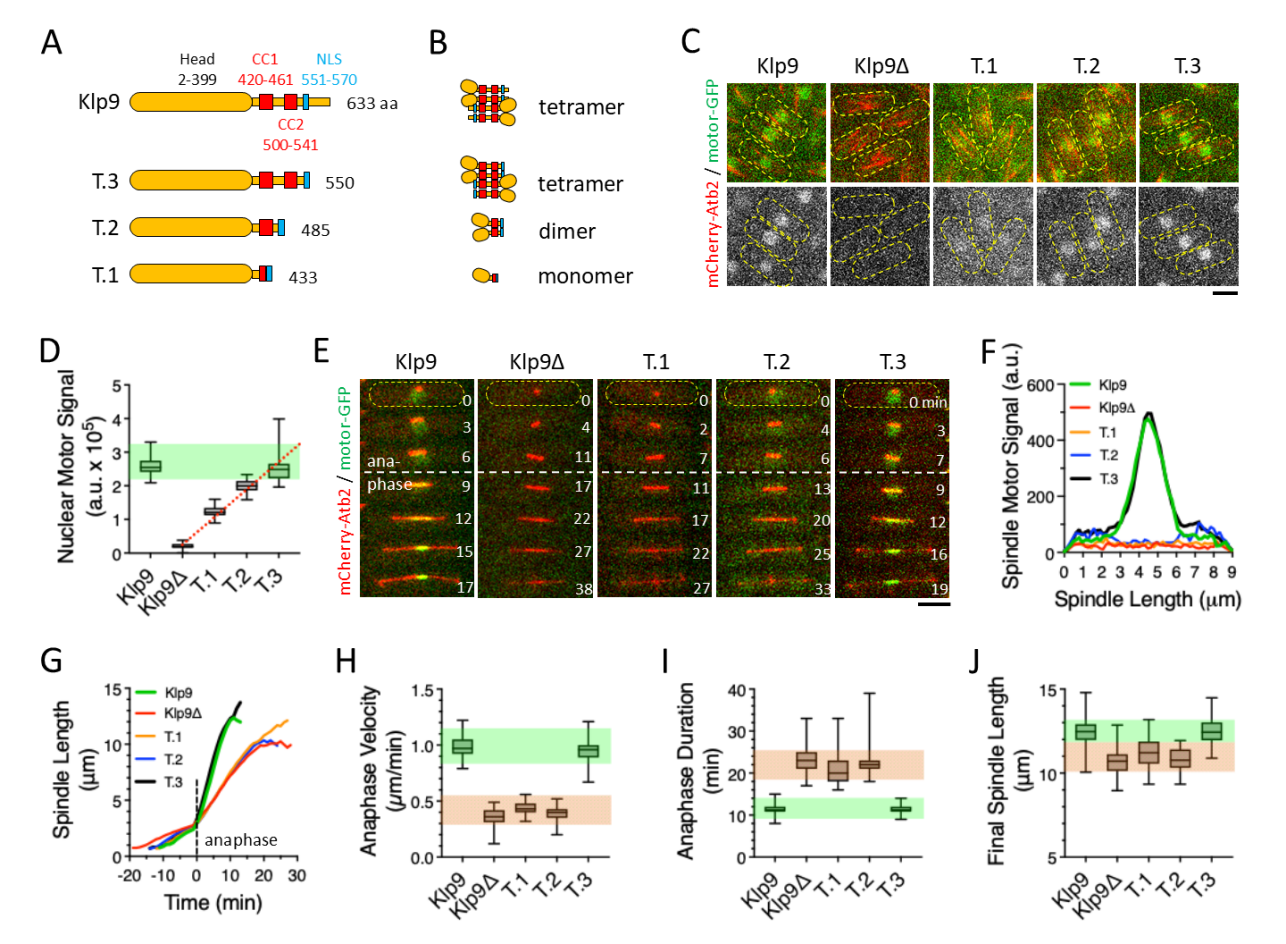
**(A)** Cartoon highlights structural domains and their amino acid position of the fission yeast kinesin-6 Klp9 . The motor head is at the N-terminus, followed by coiled-coil CC1, coiled-coil CC2, and a nuclear localization sequence NLS at the C-terminus. **(B)** Cartoon of AlphaFold3 prediction of potential oligomerization states for full-length Klp9 and its truncations, where the coiled-coils are systematically removed. Full-length Klp9 and T.3 are predicted to be antiparallel tetramers, and T.2 and T.1 are predicted to be dimer and monomer, respectively. **(C)** Spinning disk confocal fluorescent images of fission yeast cells expressing mCherry- Atb2 (tubulin) and full-length Klp9-GFP or different Klp9-truncation-GFPs. Top: merged images; Bottom: black-and-white images of motor-GFP. Cells are outlined with yellow dashed lines, with the nucleus at the cell middle. Scale = 5 µm. **(D)** Box plot of nuclear GFP signal intensity of Klp9 predicted oligomerization state. The sum intensities of n=35 nuclei were measured for each strain. Green area highlights similarity between full-length Klp9 and T.3 (tetramer). Red dotted line shows the linear regression line for T.1, T.2, and T.3 fitted to y = 0.6x + 0.5, r ^2^ = 0.8. **(E)** Time-lapsed spinning disk confocal fluorescent images of spindle dynamics of fission yeast cells expressing mCherry- Atb2 (tubulin) and full-length Klp9-GFP or different Klp9-truncation-GFPs. Dotted line delineates the start of anaphase elongation. Scale = 5 µm. **(F)** Line-scan plot of mean spindle GFP intensity signal for different predicted Klp9 oligomeric states. Each line represents mean value for n=35 spindles, measured at 9-µm length for consistency. **(G)** Plot of mean spindle length versus time for different predicted Klp9 oligomeric states. Each line represents mean length for n=35 spindles measured through mitosis. Dotted line delineates the start of anaphase elongation. **(H)** Box plot of anaphase spindle elongation velocity for different predicted Klp9 oligomeric states. Green area highlights similarity between Klp9 and T.3 (tetramer); red area highlights similarity between Klp9Δ (deletion), T1 (monomer), and T.2 (dimer). **(I)** Box plot of anaphase duration for different Klp9 oligomeric states. Green area highlights similarity between Klp9 and T.3 (tetramer); red area highlights similarity between Klp9Δ (deletion), T1 (monomer), and T.2 (dimer). **(J)** Box plot of final spindle length at the end of anaphase for different Klp9 oligomeric states. Green area highlights similarity between Klp9 and T.3 (tetramer); red area highlights similarity between Klp9Δ (deletion), T1 (monomer), and T.2 (dimer).

## Description

The fission yeast kinesin-6 Klp9 has been well studied (Fu et al., 2009; Kruger et al., 2019; Kruger et al., 2021; Yukawa et al., 2029). It functions at the spindle midzone as a tetrameric microtubule plus end-directed motor, where it binds to antiparallel microtubules and produces sliding forces to effectively elongate the spindle to separate the sister chromosomes at anaphase. In fission yeast, Klp9 is constitutively expressed and localizes in the nucleus, and binds to the spindle microtubules only during anaphase via CDK1-dependent phospho-regulation (Fu et al, 2009). In addition to the motor head at the N-terminus, the secondary structure of Klp9 contains two alpha-helical coiled-coil domains (CC1 aa 420-461, CC2 aa 500-541) and a nuclear localization sequence (NLS aa 551-570) ( Fig. 1A ) (Yukawa et al., 2019). Coiled-coils have been well-implicated in facilitating protein-protein interactions (Burkhard et al., 2001; Watkins et al., 2015). Therefore, we hypothesized that CC1 and CC2 function to dimerize and tetramerize Klp9 , respectively. To test this hypothesis, we performed predictive oligomerization states for Klp9 CC1 and CC2 using AlphaFold3, then performed confirmative structure-function live-cell experiments for Klp9 CC1 and CC2.

AlphaFold has emerged as a powerful artificial intelligence engine to predict protein structures (Jumper et al., 2021; Varadi et al., 2024) and protein-protein interactions (Abramson et al., 2024). We first analyzed the secondary structure of Klp9 to identify positions of coiled-coils ( www.pombase.org ). We then made *in silico* truncations of Klp9 full-length (aa 1-633): T.3 (aa 1-550, CC1 and CC2 present), T.2 (aa 1-485, CC1 present), and T.1 (aa 1-433, no CCs), and provided the sequences to AlphaFold Server ( www.alphafoldserver.com ), to query for potential oligomerization states ( Fig. 1A ). AlphaFold3 predicted the full-length Klp9 to be an antiparallel tetramer, T.1 a monomer, T.2 a dimer, and T.3 an antiparallel tetramer ( Fig. 1B ). We note that in cells the full-length Klp9 functions as an antiparallel tetramer (Fu et al., 2009). Thus, AlphaFold predicted correctly the oligomeric state of full-length Klp9 *in silico* . The other predictions by AlphaFold require experimental verification.

AlphaFold prediction for T.1 (monomer), T.2 (dimer), and T.3 (tetramer) enables two precise *in vivo* experimental tests to confirm: 1) The fluorescent signal of a protein attached to GFP scales linearly with the number of proteins within an optical point-spread-function, in ideal conditions (Chen et al., 2003; Dunsing et al., 2018; Vámosi et al., 2016), i.e., the signal of full-length Klp9-GFP = T.3 (tetramer) > T.2 (dimer) > T.1 (monomer); and 2) Only T.3, as a potential antiparallel tetramer, can localize to the midzone and slide antiparallel microtubules apart to elongate the spindle, similar to full-length Klp9; whereas T.1 and T.2, potential monomer and dimer, respectively, cannot slide apart antiparallel microtubules and thus spindle elongation would be attenuated, similar to Klp9-deletion.

To test these predictions, we inserted at the fission yeast Klp9 endogenous locus with its endogenous promoter, different truncations of Klp9 ( Fig. 1A ), tagged at the C-terminus with GFP, and mindful to include the NLS sequence in each truncation to ensure proper nuclear localization (Bähler et al., 1998). In addition, cells also express mCherry- Atb2 (tubulin) at its endogenous locus, to visualize the microtubule spindle. Spinning disc confocal fluorescent time-lapsed imaging (Tran et al., 2004) revealed both motor signal intensity and spindle dynamics through the cell cycle. Figure 1C shows images of interphase cells. Full-length Klp9-GFP was observed within the interphase nucleus ( Fig. 1C ), consistent with its known reported localization (Fu et al., 2009). Further, while Klp9-deletion (Klp9Δ) showed no nuclear signal, the T.1, T.2, and T.3 truncations showed successive increase in nuclear signal ( Fig. 1C ), consistent with the potential increasing oligomeric state of Klp9-truncations. Signal intensity analyses revealed a positive linear correlation between Klp9 and its potential increasing oligomeric state ( Fig. 1D ): linear regression analysis fitted y = 0.6x + 0.5, slope = 0.6, r ^2^ = 0.8; where y is the intensity signal and x is the oligomeric state of the motor. In ideal conditions, the signal of two GFPs should be 2X higher than one GFP, and four GFPs should be 4X higher than one GFP, etc., leading to a regression slope of 1. In experimental conditions, this scaling correlation is expected to be less than 1 with increasing GFP number, due to optical defects, the energy quenching effects of adjacent GFPs, and the cellular milieu (Chen et al., 2003; Dunsing et al., 2018; Vámosi et al., 2016). Our result showed that T.3 has the same signal as full-length Klp9 : Klp9 2.6 ± 0.3 x10 ^5^ a.u.(n=35) versus T.3 2.5 ± 0.4 x10 ^5^ a.u. (n=35), p=0.45, indicating that like full-length Klp9 , T.3 is a tetramer. Further, the signal of T.3 (2.5 ± 0.4 x10 ^5^ a.u.(n=35)) > T.2 (2.0 ± 0.2 x10 ^5^ a.u.(n=35), p<0.001) > T.1 (1.2 ± 0.2 x10 ^5^ a.u.(n=35), p<0.001) scales linearly with slope = 0.6 ( Fig. 1D ), indicating that T.2 is likely a dimer, and T.1 likely a monomer. Thus far, signal intensity analyses of Klp9 and its truncations suggest different oligomeric states consistent with the AlphaFold prediction.

We next measured spindle dynamics for the Klp9 series. Spindle elongation in fission yeast occurs in three phases: prophase/metaphase, metaphase, and anaphase, each with distinct spindle lengths, durations, and velocities (Nabeshima et al., 1998). Klp9 is nuclear throughout interphase up to metaphase. At anaphase, Klp9 binds to the spindle, moves in a microtubule plus end-directed manner toward the spindle midzone, and produces sliding forces to separate the antiparallel microtubules, thus elongating the anaphase spindle (Fu et al., 2009). The absence of Klp9 attenuates anaphase spindle elongation (Fu et al., 2009). Figure 1E shows time-lapsed images of mitotic cells. Full-length Klp9-GFP and T.3 both localized to the spindle midzone at anaphase ( Fig. 1D ). In contrast, T.1 and T.2 did not localize to the spindle midzone ( Fig. 1D ). Further, signal line-scan analyses revealed that both Klp9 and T.3 bind to the spindle midzone at the same mean intensity or amount ( Fig. 1E ): Klp9 6.2 ± 1.4 x10 ^3^ a.u. (n=35) versus T.3 6.3 ± 1.6 x10 ^3^ a.u. (n=35), p=0.77. Importantly, signal line-scan analyses indicated that T.1 and T.2, like Klp9Δ, do not bind to the spindle midzone ( Fig. 1E ). This result implies that spindle dynamics of T.3 are likely to be similar to full-length Klp9; and that spindle dynamics of T.1 and T.2 are likely to be similar to Klp9Δ. Indeed, spindle dynamic measurements revealed that Klp9 and T.3 have near identical mean spindle elongation curves ( Fig. 1G ); anaphase elongation velocities (Klp9 0.98 ± 0.1 µm/min (n=35) versus T.3 0.94 ± 0.1 µm/min (n=35), p=0.08) ( Fig. 1H ); anaphase durations (Klp9 11 ± 1 min (n=35) versus T.3 11 ± 1 min (n=35), p=0.7) ( Fig. 1I ); and final spindle lengths (Klp9 12.42 ± 0.92 µm (n=35) versus T.3 12.54 ± 0.77 µm (n=35), p=0.53) ( Fig. 1J ). In contrast, while Klp9Δ, T.1, and T.2 are different from full-length Klp9 and T.3, they are similar to each other. Spindle dynamic measurements revealed Klp9Δ, T.1, and T.2 have similar spindle elongation curves ( Fig. 1G ); anaphase elongation velocities (Klp9Δ 0.36 ± 0.07 µm/min (n=35) versus T.1 0.44 ± 0.06 µm/min (n=35), p<0.001, versus T.2 0.39 ± 0.07 µm/min (n=35), p=0.03) ( Fig. 1H ); anaphase durations (Klp9Δ 23 ± 3 min (n=35) versus T.1 21 ± 4 min (n=35), p<0.01, versus T.2 22 ± 3 min (n=35), p=0.14) ( Fig. 1I ); and final spindle lengths (Klp9Δ 10.72 ± 0.82 µm (n=35) versus T.1 11.23 ± 0.88 µm (n=35), p<0.05, versus T.2 10.78 ± 0.64 µm (n=35), p=0.75) ( Fig. 1J ). The result indicates that T.3 is likely a tetramer, because like full-length Klp9, it localizes to the spindle midzone and elongates the spindle, with similar dynamics. In contrast, T.1 and T.2 are likely monomer and dimer, respectively, because while they are expressed in cells, they cannot perform spindle elongation, similar to Klp9Δ. Again, the spindle dynamic analyses for Klp9 and its truncations also suggest oligomeric states consistent with AlphaFold prediction. We note that the unstructured C-terminal aa551-633, absent from T.3, appeared not important for its function in spindle localization and elongation.

In summary, fission yeast kinesin-6 Klp9 is a molecular motor, which functions as a tetramer that slide antiparallel microtubules at the spindle midzone apart (Fu et al., 2009; Krüger et al., 2019; Krüger et al., 2021; Yukawa et al., 2019). Klp9 contains two coiled-coils domains CC1 and CC2, and coiled-coils are known to facilitate protein-protein interactions. AlphaFold3 predicted that CC1 is involved in the dimerization of Klp9 , and CC2 (together with CC1) is involved in antiparallel tetramerization of Klp9 . We then performed experiments in fission yeast cells expressing full-length Klp9 and Klp9-truncations that removed CC2 or both CC1 and CC2. Our data revealed that Klp9-truncation lacking CC2 shows fluorescent signal and spindle dynamics consistent with being a dimer, Klp9-truncation lacking both CC1 and CC2 shows fluorescent signal and spindle dynamics consistent with being a monomer. Only Klp9-truncation having both CC1 and CC2 shows fluorescent signal and spindle dynamics consistent with being an antiparallel tetramer. We conclude that fission yeast Klp9 CC1 domain functions to dimerize Klp9, and CC2 (together with CC1) functions to antiparallelly tetramerize Klp9. Our work highlights the synergy between artificial intelligence-aided prediction using AlphaFold combined with cellular experimental confirmation.

## Methods

Yeast strains and media:

We employed standard yeast genetic techniques to create and maintain the strain utilized in this study (Moreno et al., 1991). Typically, cells were cultured on agar plates containing YE5S media at a temperature of 25⁰C.

For microscopy experiments, a small number of cells were inoculated into liquid YE5S medium the night before the experiment. The culture was then incubated at 25⁰C with continuous shaking overnight until it reached an optical density OD _600nm_ ≈ 0.5 absorbance units. At this point, the cells were harvested for imaging. The cells were mounted in YE5S media within 2% agarose using custom-made PDMS chambers, as previously described (Costa et al., 2013).

Microscopy:

Imaging was conducted using a spinning disk confocal microscope setup (Tran et al., 2004). Specifically, we utilized a Nikon Eclipse Ti2 inverted microscope equipped with a Nikon CFI Plan Fluor 100x/1.4 NA objective lens, a Nikon Perfect Focus System (PFS), a Mad City Labs integrated Nano-View XYZ micro- and nano-positioner, a Yokogawa Spinning Disk CSU-X1 unit, a Photometrics Cascade EMCCD camera, and a Gataca Systems solid-state laser unit with 488 nm (10 mW) and 561 nm (10 mW) lines, controlled by Molecular Devices MetaMorph 7.0 software. The microscope was enclosed inside a Life Imaging Services thermal box, with temperature controlled via forced air from the thermal Cube. The temperature was maintained stably at the set point 25 ± 1⁰C.

To capture the images, camera settings were set at EM-Gain: 300 and Gain: 3X. We acquired z-stacks consisting of 7 focal planes spaced 1 µm apart in the GFP (Exposure time: 100 ms) and mCherry (Exposure time: 200 ms) channels. A corresponding brightfield image (Exposure: 50 ms) was acquired to visualize cells. The stacks were acquired through time-lapse at 1 min intervals, for 75 min total time

Analysis:

For nuclear GFP intensity measurements, the sum-projection of seven optical sections, covering the complete cell and its nucleus, was visualized and analysed by ImageJ/FIJI (Schindelin et al., 2012). For spindle length measurements, maximum-projection time-lapsed movies were visualized and analysed by ImageJ/FIJI (Schindelin et al., 2012). Statistical analysis, e.g., mean ± s.d., linear regression, t-test, were performed using GraphPad Prism. Plots were generated using GraphPad Prism.

## Reagents

Strains:

TP.5943 h- Klp9-FL(aa1-633)-GFP:KanR mCherry- Atb2 :HygR

TP.5158 h- Klp9Δ:NatR mCherry- Atb2 :HygR

TP.5946 h- Klp9-T.1(aa1-433)-GFP:KanR mCherry- Atb2 :HygR

TP.5947 h- Klp9-T.2(aa1-485)-GFP:KanR mCherry- Atb2 :HygR

TP.5948 h- Klp9-T.3(aa1-550)-GFP:KanR mCherry- Atb2 :HygR
